# Excessive fluid balance effect in mortality rate from surgical patients

**DOI:** 10.1186/cc12673

**Published:** 2013-06-19

**Authors:** JM Silva, FAM Nogueira, PMM Vianna, MCP Filho, CS Neucamp, VPL Maia, AMRR de Oliveira, MJC Carmona, LM Sá Malbouisson

**Affiliations:** 1 Hospital das Clinicas, Cerqueira Cesar, São Paulo, SP, Brazil

## Introduction

In some studies including small populations of patients undergoing gastrointestinal surgery, a liberal infusion of fluids during the intraoperative period was associated with increased morbidity when compared with restrictive strategies for volume replacement. However, there are few studies evaluating the role of excessive fluid infusion in a population of high-risk surgical patients intraoperatively. The aim of this study was to evaluate the impact of the excessive water balance in the perioperative morbidity and mortality of high-risk surgical patients.

## Methods

A prospective cohort study during 1 year in four ICUs from three tertiary hospitals. The study included patients who required postoperative ICU, aged ≥18 years undergoing major surgery. Patients with palliative surgery were excluded. The calculation from fluid balance was based on the surgical duration, the time of fasting preoperative and intraoperative, urine output and fluid replacement intraoperatively.

## Results

The study included 479 patients. Mean age was 61.2 ± 17.0 years and 51.1% were men. Nonsurvivors in the hospital represented 8.8% of patients. The median duration of surgery was 4.0 (3.2 to 5.5) hours. The average value of the SAPS 3 score was 41.8 ± 14.5 and ASA II was 52.8%. However, when comparing survivors and nonsurvivors, the fluid balance intraoperatively from nonsurvivors was higher (1,950 (1,400 to 3,400) ml vs. 1,400 (1,000 to 1,600) ml, *P *<0.001), patients with fluid balance above 2,000 ml intraoperatively had longer ICU stay (4.0 (3.0 to 8.0) vs. 3.0 (2.0 to 6.0), *P *= 0.000) and higher incidence of infectious complications (41.9% vs. 25.9%, *P *= 0.001), neurological (46.2% vs. 13.2%, *P *= 0.000), cardiovascular (63.2% vs. 39.6%, *P *= 0.000) and respiratory (34.3% vs. 11.6%, *P *= 0.000). In multivariate analysis, the fluid balance was an independent factor for death (OR = 1.002, *P *= 0.04, 95% CI = 1.001 to 1.004). See Table 1.

## Conclusion

Patients with excessive fluid balance intraoperatively have more ICU complications and higher hospital mortality.

**Table 1:** Independent variables of hospital death

			95% Cl	
			
	*P *value	OR	Lower	Upper
Males	0.170	1.697	0.797	3.610
SAPS 3	0.002	1.051	1.019	1.084
ASA	0.010	1.728	1.143	2.613
Intraoperative blood transfusion	0.645	1.210	0.539	2.715
Fluid balance intraoperatively (ml)	0.042	1.002	1.001	1.004
Vasopressors intraoperatively	0.328	1.531	0.652	3.596

Variables included in the model: sex, SAPS 3, ASA, transfusion, fluid balance intraoperatively, intraoperative vasoactive drugs.

